# On the quantification of local power densities in a new vibration bioreactor

**DOI:** 10.1371/journal.pone.0245768

**Published:** 2021-01-22

**Authors:** David Valentin, Alexandre Presas, Charline Roehr, Elisa Mele, Christoph Biehl, Christian Heiss, Wolfram A. Bosbach

**Affiliations:** 1 Center for Industrial Diagnostics and Fluid Dynamics (CDIF), Polytechnic University of Catalonia (UPC), Barcelona, Spain; 2 Experimental Trauma Surgery, Justus-Liebig University of Giessen, Giessen, Germany; 3 Materials Department, Loughborough University, Loughborough, United Kingdom; 4 Department of Trauma, Hand and Reconstructive Surgery, University Hospital of Giessen, Giessen, Germany; University of Notre Dame, UNITED STATES

## Abstract

We investigate the power densities which are obtainable locally in a vibration bioreactor. These reactor systems are of great relevance for research about oncological or antibacterial therapies. Our focus lies on the local liquid pressure caused by resonance vibration in the fluid contained by the reactor’s petri dish. We use for the excitation one piezoelectric patch which offer advantages concerning controllability and reproducibility, when compared to ultrasound. The experimental work is extended by finite element analyses of bioreactor details. The peaks of the vibration response for water, sodium chloride (0.1N Standard solution), and McCoy’s 5A culture medium are in good alignment. Several natural frequencies can be observed. Local power density can reach multiple times the magnitude used in ultrasound studies. Based on the observed local power densities, we are planning future work for the exposure of cell cultures to mechanical vibration.

## 1. Introduction

The biological phenomena inside human cells under mechanical vibration are not as well understood as they should be. One challenge on the way towards an improved understanding of this is the study of metabolic responses exhibited by the cells. To do so, efficient vibration bioreactors are necessary. A suitable reactor system will allow for studying cellular behaviour, and the involved metabolic processes, while controlling reproducibly vibration actuation patterns. In our paper here, we investigate the migration of vibration into petri dishes and culture medium. The investigations build up on our previous work on structural vibration responses [1–3] and cell mechanics [4, 5].

The long term objective of our ongoing work is to make vibration phenomena accessible for novel vibration therapies in a medical, clinical setting. Two medical fields which could take advantage of the study in bioreactors in this present paper are oncological therapies and antibacterial measures against biofilm forming bacteria. In both, literature has found cell responses which we deem worthwhile for further study.

In trauma and orthopaedic surgery, the most frequent malignant bone neoplasm is the Osteosarcoma (OS), followed by the Chondrosarcoma and the Ewing sarcoma [6]. Treatment of the OS by vibration has been tested in the past by a procedure known as high intensity focused ultrasound (HIFU) [7, 8]. HIFU is also in the discussion for the treatment of liver or kidney tumours [9]. Results so far have been unfortunately unsatisfying; although the basic desired effect of OS cell destruction is achievable for power densities of up to 300 W/cm^2^ [7, 8, 10, 11]. The combination of HIFU technology with magnetic resonance guidance [12] for the sake of greater controllability of the intervention is a matter of ongoing clinical research. Interestingly, *in-vitro* tests with OS cells (Saos-2 cell line) at far lower power densities of around 30 mW/cm^2^ at frequencies of 1.5 MHz [13–16] respond by enhanced cell proliferation, in contrast to the *in-vivo* cell destruction under HIFU mentioned above. For both types of studies, the intracellular processes taking places are still not understood fully. The basic effect of bone reacting to vibration has been shown in 1984 already and has been subject of our previous work [4, 5, 17, 18]. One great shortcoming of the existing HIFU method is that ultrasound is an indirect way of mechanical excitation. This means that frequency and intensity of the vibration are difficult to control which leads to only a limited reproducibility of the experimental observation.

Biofilm forming bacteria which are the second focus of this present study are a particular threat to patient outcome in trauma and orthopaedic surgery; and of course also in other medical specialties. Any foreign materials (implanted joints, catheters, pacemakers) can be colonised by biofilm forming bacteria in clinical application. The two dominant species of greatest relevance in trauma and orthopaedic surgery are *Staphylococcus Aureus* (SA) and *Staphylococcus Epidermidis* (SE) [19, 20]. Adhesion surfaces inside a patient offer these both an opportunity for increased pathogenicity. Proliferating eg on suture material, SA achieves greater infection rates than without it [21]. It uses on top specific proteins such as protein A, or surface proteins A to K [22]. In a highly interesting experiment, it has been possible to specifically link genes responsible for the building of bacterial adhesion organelles to occurrence rates of clinical infections. *Escherichia coli* (EC) assembles through the papG gene so called P fimbriae which increase the occurrence rate of urinary tract infection [23]. Because of their documented pathogenicity, adhesion organelles are one of our future focus topics. The morphology of adhesion surfaces is known to greatly influence biofilm forming further [24, 25]. Also for the treatment of biofilms, ultrasound is being tested. *In-vitro* and *in-vivo* studies exist [26, 27] where the effect is tested for a combination with antibiotic pharmacological therapy.

Research has shown for both of the two above examples, OS and biofilm forming bacteria, that vibration can be used to achieve beneficial effects. The limitations of mechanical excitation by ultrasound are the motivation for our work. We see the potential for further improvements from resonance excitation which allows for greater process controllability and better experimental reproducibility. With the tested bioreactor, *in-vitro* studies may be performed to see the effects of vibration in cells. After the quantification of those effects in cells, if they are promising, further studies should analyse how to apply these vibration levels to organisms.

In the text following below, we present the tested vibration bioreactor dimensions and obtained vibration characteristics for the contained liquid media (water, sodium chloride 0.1N standard solution, McCoy’s 5A w/L-Glutamine culture medium). These three media were chosen to study besides water the influence on the vibration response of electrolytes and of substrates added to culture media. The discussion compares this work to ultrasound studies [13–16, 26–28]. A similar reactor geometry has been used recently by a different research group working independently from us for experiments with mesenchymal stem cells [29].

## 2. Methodology

This study tests the vibration characteristics of a vibration bioreactor design. In a previous study [1], the proof of concept of resonance vibration was presented. In this study now, the vibration transmission to the liquid medium is studied and quantified. To do so, both experiments and numerical simulations are necessary. With the experiments, vibration is applied to a petri dish containing three different liquids: water, sodium chloride 0.1N standard solution and McCoy’s 5A w/L-Glutamine culture medium. The vibration transmitted to the liquid is measured experimentally in one-single point. To know the vibration pattern of the whole liquid media and not only on a single point, numerical simulations are used. The numerical model is feed with the same vibration amplitude input than in the experiment and with the damping obtained experimentally. In this way, the local power densities provided by the vibration bioreactor in the whole liquid medium can be determined.

### 2.1. Experimental investigation

#### 2.1.1. Experimental apparatus

**Fig 1:**
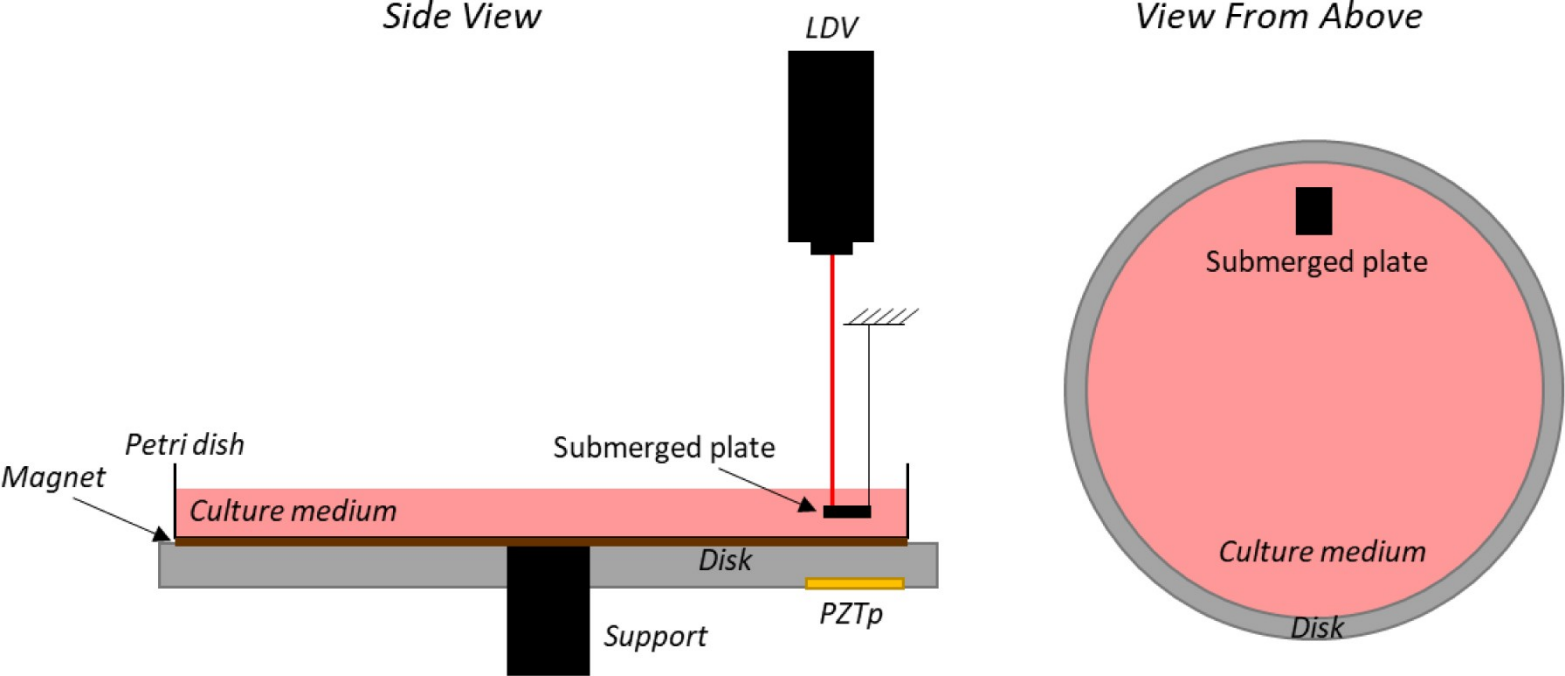
Vibration bioreactor sketch with PZTp, LDV, and submerged plate inside the culture medium.

A bronze disk (145 mm diameter and 6 mm thickness) supported at its centre and with a glass petri dish (120 mm diameter) attached to it with a magnet is used. Two pieces of magnet (same diameter than the petri dish and 0.5 mm thickness) were glued to the bottom of the petri dish as well as on the disk. The dimensions are selected to be similar to those ones of a petri dish commonly used in the laboratory. The disk is excited by a Piezoelectric Patch (PZTp, P-876 DuraAct, see Fig 1) which is glued to the disk surface with an epoxy component LOCTITE454. The petri dish is filled with three different liquids previously stated. For the experiments, the petri dish is filled to a depth of 10 mm. A small aluminium plate (8.0 mm × 6.0 mm × 0.5 mm) is submerged in the petri dish at a depth of 5 mm (see Fig 1) in order to measure the vibration in it using a Laser Doppler Vibrometer (LDV). In that way, the vibration that the culture medium will be subjected to is directly measured inside the fluid. The aluminium plate is supported by a thin wire which permits its free vibration.

### 2.2. Procedure

The excitation method follows two steps. The first step is to determine the natural frequencies of the disk when the petri dish is filled with one of the three liquids. For that, a chirp excitation from the PZTp is used (0 to 22 kHz) (-4 to 10V). The response is measured by the LDV pointed directly at the disk surface. Five different chirps were done and the average of them was used for the analysis. Coherence was also plotted to ensure the repeatability of every chirp.

Once the natural frequencies of the disk are determined, the second step is to excite the disk in different natural frequencies and to measure the response of the submerged aluminium plate by the LDV (14V peak to peak). With this procedure, the vibration that the cells will be exposed to is measured for different frequencies. Three natural frequencies in different frequency ranges are selected for this purpose. For every natural frequency, five excitations were done and the average of them was used for the analysis. Again, as in the case of the chirps, coherence was plotted. The natural frequencies of the medium itself have not been calculated, neither sloshing modes of it. As we only focused on the vibration transmitted to the cells at the disk natural frequencies, the dynamic behaviour of the medium was not studied.

### 2.3. Numerical simulation

Additionally, a numerical model is used in this study. For the calibration of the PZTp, Presas et al. [2] used a numerical model to estimate the force given by the PZTp, since this force is totally dependent on the frequency of excitation. In the numerical model, the only required input is the damping of each natural frequency which can be extracted from the experiment. This exact procedure is also followed in this paper for the used excitation by PZTp. Once the numerical model is validated, it is also useful for detailed analyses about mode-shapes and the transmission into the liquid.

**Fig 2:**
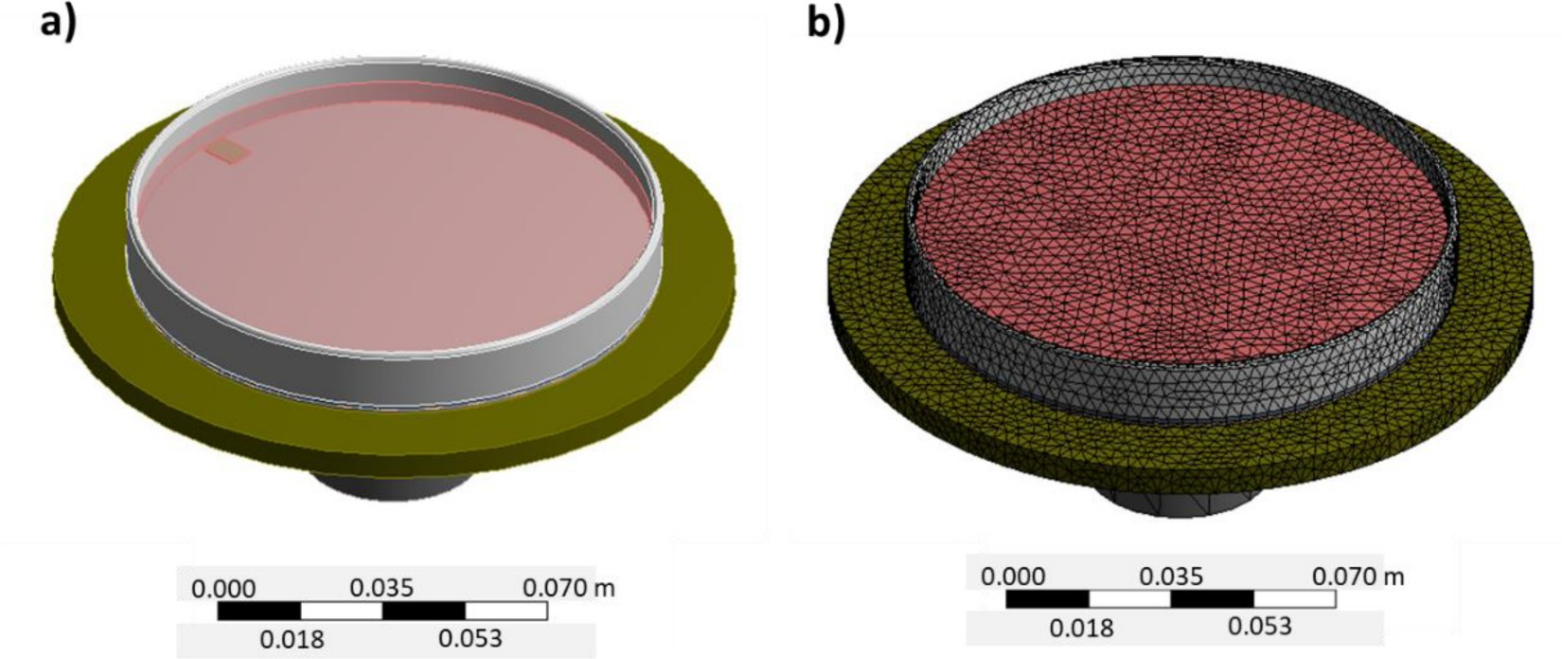
a) Geometry as computer aided drawing and b) finite element mesh used for the numerical model in Ansys v18 [31].

The numerical model considers the disk (ρ = 7666 kg/m^3^, E = 10^11^ Pa), the support (ρ = 7800 kg/m^3^, E = 2·10^11^ Pa), the petri dish (ρ = 2500 kg/m^3^, E = 7·10^10^ Pa), the liquid (ρ = 1000 kg/m^3^), and the aluminium plate (ρ = 2700 kg/m^3^, E = 7·10^10^ Pa). The petri dish and the disk are joined using a frictionless connection to model the magnet. The acoustical formulation was used to model the liquid [30]. Since this model is inviscid, only the density of the liquid and its speed of sound is simulation input. Both modal and harmonic analysis are performed using the commercial software Ansys v18. The sensitivity to mesh size was checked in the previous work [1]. The geometry and the mesh used are shown in Fig 2.

## 3. Results and discussion

### 3.1. Natural frequencies and mode-shapes

**Fig 3:**
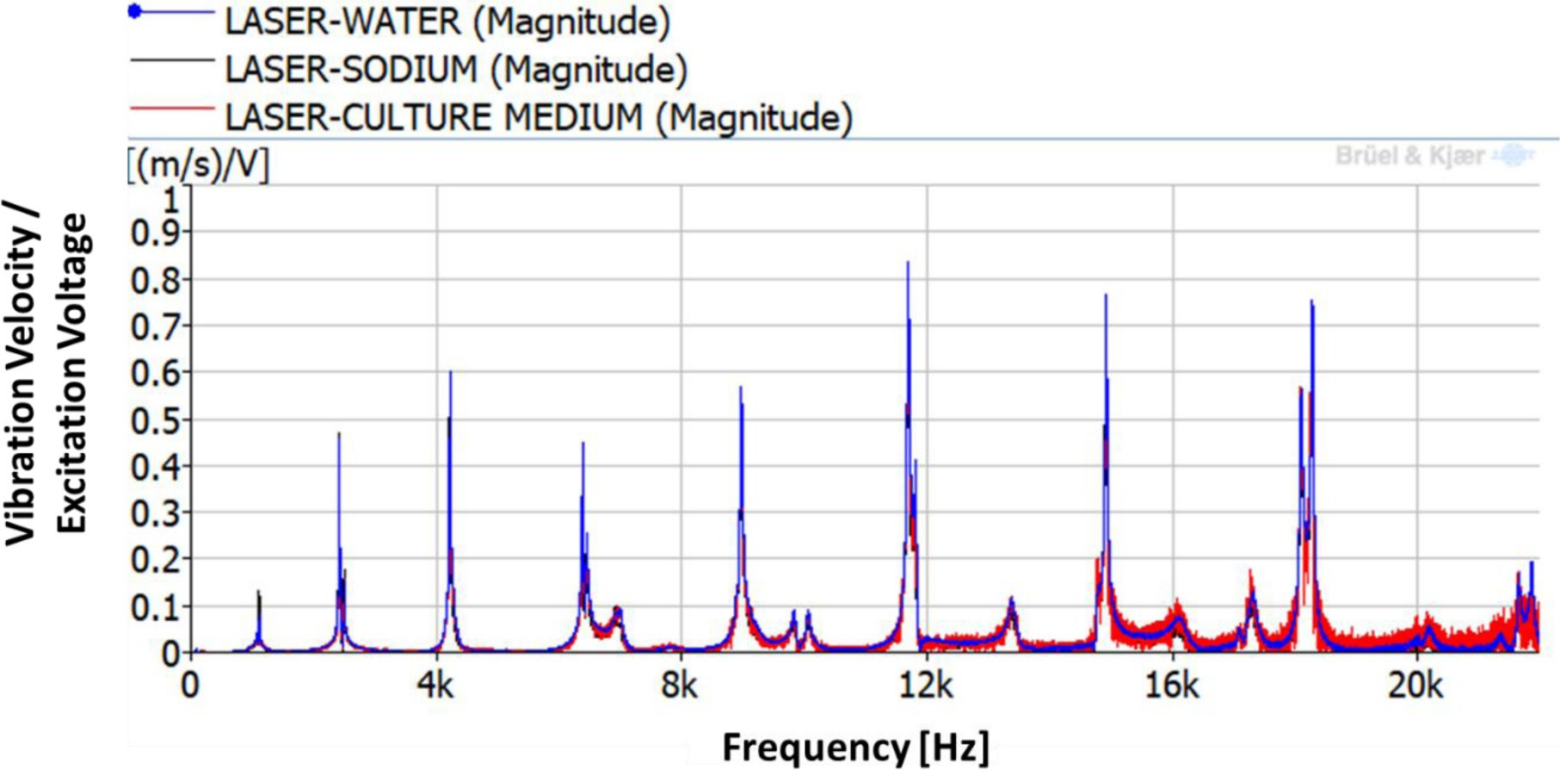
Frequency Response Function (FRF) between the LDV and the PZTp.

As explained in the methodology section, the first step is the determination of the natural frequencies of the disk with the petri dish filled with the three different liquids 10 mm deep. A chirp excitation by the PZTp is performed within the frequency range of 0–22 kHz. The results for the vibration of the disk are shown in Fig 3. The different natural frequencies of the disk can be seen in this figure. Those natural frequencies are hardly changed when compared to the ones obtained in the previous work [1] without a petri dish (see Table 1 for further details). The mass added to the disk by the petri dish and by the liquids is negligible with regard to the study’s investigations. The magnet is not adding stiffness to the disk. Consequently, the mode-shapes of the dish are unchanged to the ones for the disk in air, with the difference that they now can provide displacement to the petri dish and subsequently vibration to the liquid. Those mode-shapes can be analysed further by means of the numerical modelling (see below).

**Table 1:** Experimental values of natural frequencies in comparison to those ones obtained without petri dish.

Mode-shape	Without pretri dish f [Hz] [1]	With petri dish		
		Water f [Hz]	Sodium f [Hz]	Culture medium f [Hz]
4ND	4197	4219	4216	4221
6ND	8982	8972	8967	8968
8ND	14929	14921	14914	14913

**Fig 4:**
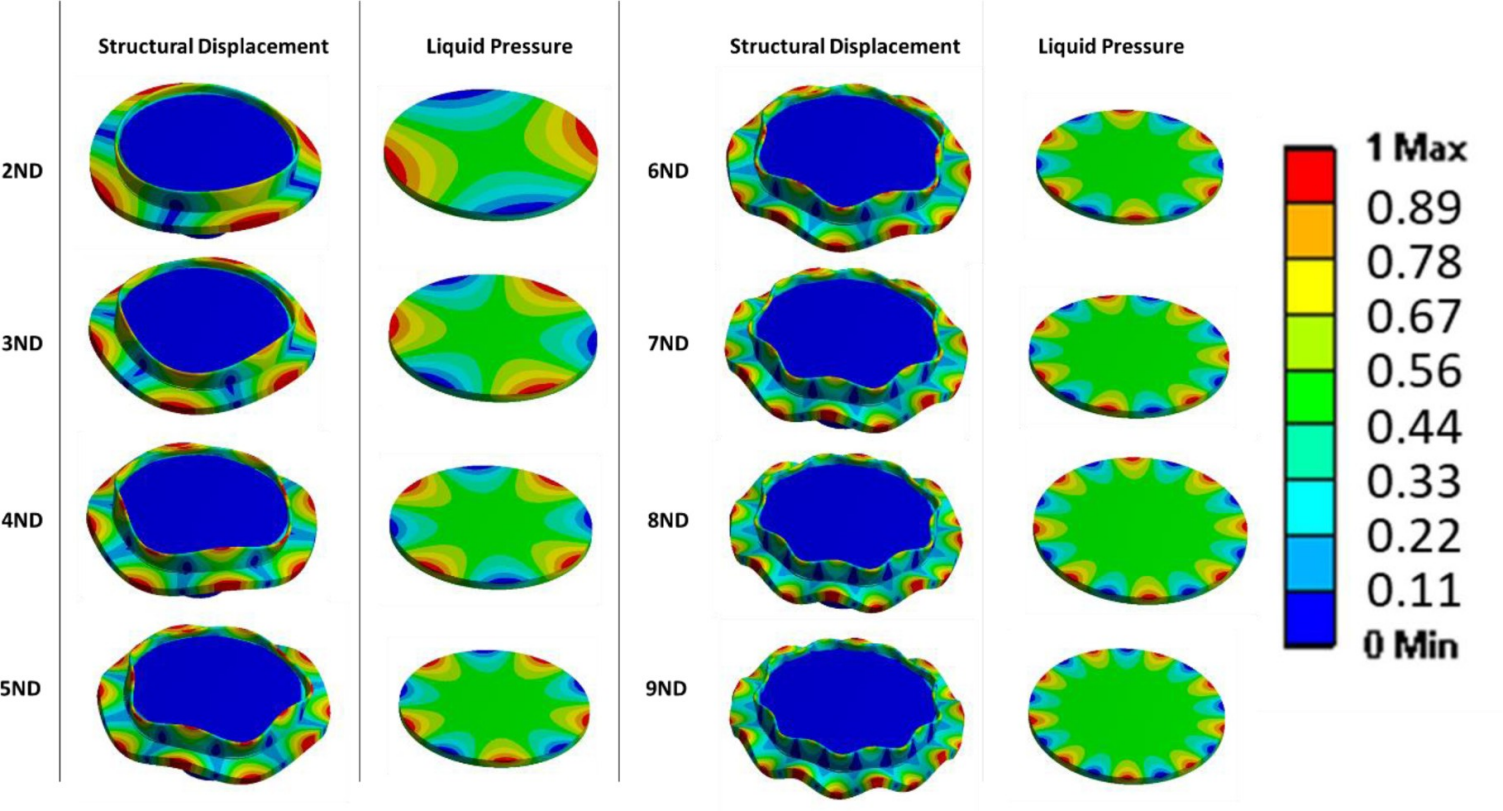
Mode-shapes of the disk together with the petri dish (displacement), and pressure in the liquid due to the vibration of the disk. Colours are related to relative displacements and pressure, since it is a modal analysis (no force applied), both displacement and pressures are relative.

Fig 4 shows selected mode-shapes obtained by the numerical simulation. Only nodal diameter (ND) mode-shapes are here considered. It can be seen that for greater number of nodal diameters, the deformation is more concentrated in the periphery of the disk and that the maximum pressure in the liquid is also concentrated in the peripheral part of the petri dish. This means that in future cell testing cells located at the periphery will vibrate at greater amplitude than the ones located in the centre of the petri dish. We assume that cells will be moving as the fluid, from maximum to minimum pressure zones during the excitation, therefore the cells in the centre will remain stationary during the vibration process. This is interesting in order to compare the results of cell behaviour under vibration. The location of the cells in the petri dish will affect the behaviour under vibration greatly. In resonance mechanics, vibration frequency will be constant throughout the system. Amplitude of vibration however varies across a system under resonance. The mentioned aluminium plate is located in the exterior part of the petri dish but not in contact with the disk itself or the lower surface of the petri dish. It is expected to have lower vibration amplitudes than the disk since the vibration is transmitted by the liquid.

### 3.2. Cell vibration under forced response

**Fig 5:**
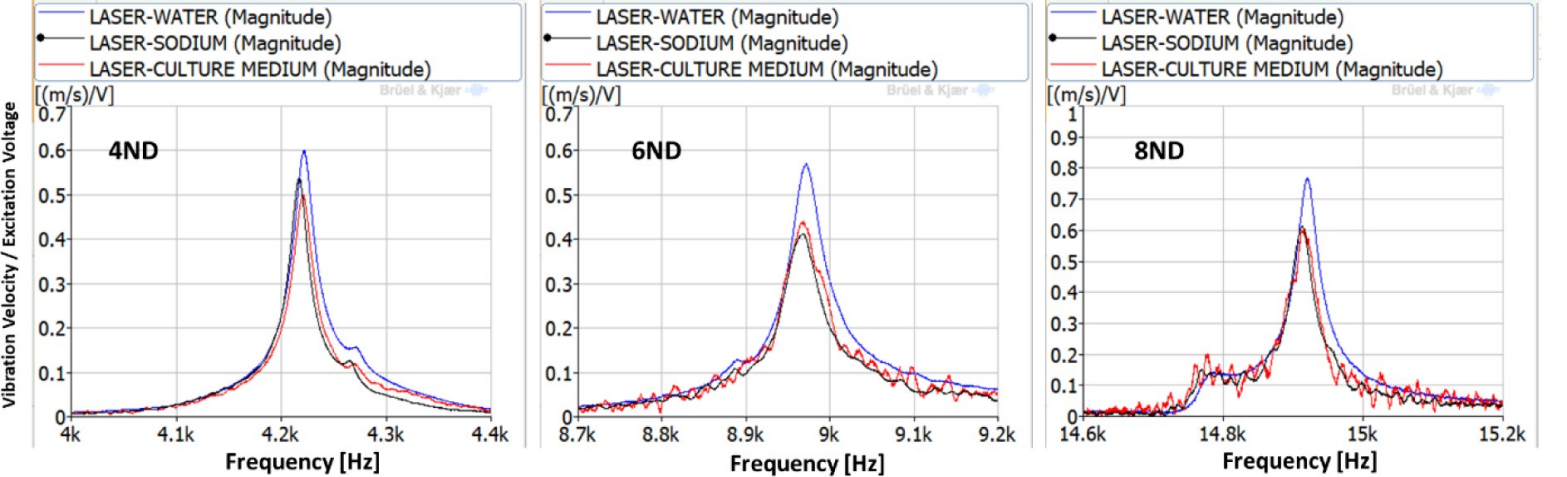
FRFs between the LDV and the PZTp for the natural frequencies selected for study (4,216–4,221Hz, 8,967–8,972 Hz, and 14,913–14,921 Hz).

For the estimation of the vibration amplitude of the cells inside the culture medium, the disk is excited for the duration of 10 seconds using three different natural frequencies. The natural frequencies selected are the ones corresponding to the 4ND, 6ND and 8ND mode-shapes, with values of 4,216–4,221Hz, 8,967–8,972 Hz and 14,913–14,921 Hz. The natural frequencies marginally change for each liquid because of the difference in liquid density, as seen in previous studies [32, 33]. These three natural frequencies for the different liquids can be seen in Fig 5. The plot in Fig 5 is a zoom of the selected natural frequencies of Fig 3.

**Fig 6:**
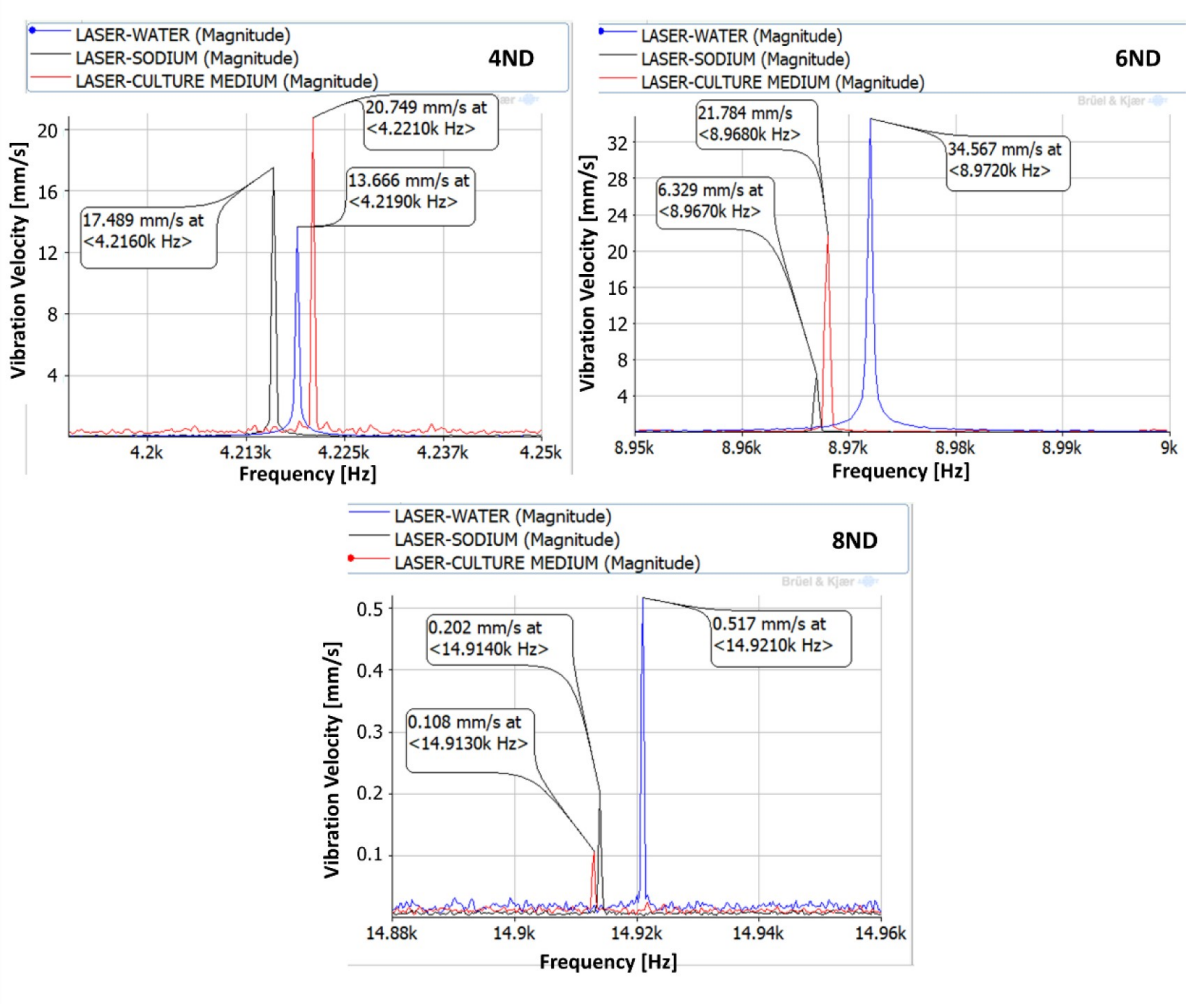
Vibration of the aluminium plate under a forced response excitation study (4,216–4,221Hz, 8,967–8,972 Hz, and 14,913–14,921 Hz).

The vibration of the aluminium plate under the forced response excitation is shown in Fig 6. The excitation voltage amplitude is defined as the maximum possible, given the amplifier characteristics. It is observed that the amplitudes of vibration are different for the three different liquids. Generally, the plate vibrates with greater amplitudes in water. While greater viscosity in the culture medium and in the sodium, presents a marginally lower vibration amplitude, similar in both those liquids. This behaviour was confirmed in [32, 33]. For the 4ND mode-shape, the amplitude of vibration inside the medium, and therefore the one that the cells will be subjected to is about 20 mm/s at a frequency of about 4,200 Hz. For 6ND mode-shape, the frequency is approximately doubled, about 8,900 Hz, and the amplitude remains in the same range (between 20–35 mm/s depending on the liquid). However for greater frequencies, as for example in 8ND which is located at about 14,900 Hz, the amplitude of vibration decreases greatly, having a maximum of 0.5 mm/s. This can be explained by Fig 4, which shows that the pressure on the disk is decreasing when increasing the number of nodal diameters in the zone where the aluminium plate is located.

### 3.3. Power density

To calculate the power density that the cells are subjected to, it is necessary to know first the force that is applied to the disk, and after that the force that is transmitted to the cells. The force of the PZTp depends on multiple parameters. The only way to establish it, once installed on the disk, is to perform a numerical structural harmonic analysis as Presas et al. [2] shown in their investigation. This harmonic structural analysis is based on the application of a force with variable magnitude and introducing the damping obtained by the experiment. Once the vibration of the structure obtained by numerical simulation corresponds to the vibration obtained experimentally, the force given by the patch is determined and the numerical simulation validated.

The results obtained after applying this procedure are shown in Table 1. With the vibration measured by the LDV in a plate submerged in the liquids, the cell vibration amplitude can be obtained as well as the force that they are subjected to. The value of this force for the different ranges of excitation is shown in Table 2. Additionally, the power densities are calculated by multiplying the force by the vibration velocity and dividing by the submerged plate surface in Table 3. As one can see, the values are generally higher for lower frequencies, the power densities of the highest frequency tested being very low. This method is based on the mechanical resonance of a disk which has relatively low values of natural frequencies, therefore the maximum vibration amplitudes are reached for these low values of frequency. The value of power density calculated for the cells in the location of the submerged plate can be extrapolated for the whole petri-dish with the culture medium trough numerical simulations.

**Table 2:** Force applied to the disk obtained by numerical simulation.

Mode-shape	Force applied to the disk (N)	Cell vibration amplitude (mm/s)		
		Water	Sodium	Culture medium
4ND	11.50	13.666	17.489	20.749
6ND	13.50	34.567	6.329	21.784
8ND	9.37	0.517	0.202	0.108

**Table 3:** Force received by the cells and power density for every case tested.

Mode-shape	Force received by the cells (N)			Power Density (W/cm^2^)		
	Water	Sodium	Culture medium	Water	Sodium	Culture medium
4ND	0.35	0.53	0.81	9.94	19.35	34.86
6ND	1.73	0.81	0.51	124.47	10.68	23.17
8ND	2.16e-2	4.66e-4	9.74e-5	2.33e-2	1.96e-4	2.19e-05

### 3.4. Comparison to other non-direct methods

**Fig 7:**
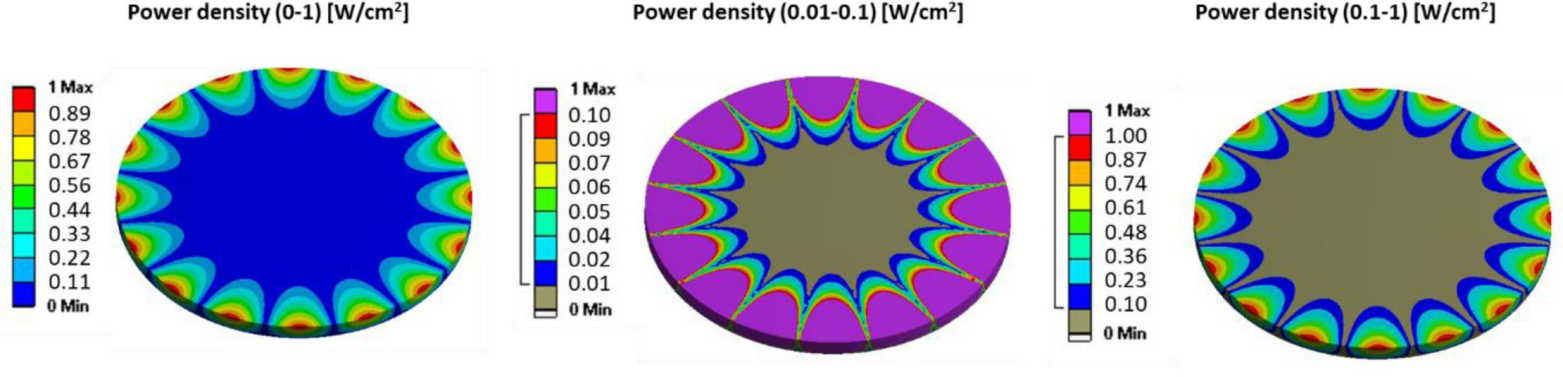
Power densities at 8ND in the petri dish for the culture medium case: 0 to 1 W/cm^2^, 0.01 to 0.1 W/cm^2^ (referring to [13–16]), and 0.1 to 1 W/cm^2^ (referring to [26, 27]).

Through the work presented above by us, the power densities which the cell cultures will be subjected to are known. These values can be compared to other non-direct methods in *in-vitro* cell studies as done in Fig 7 for the 8ND mode-shape. Ultrasound excitation has been applied on OS [13–16] at frequencies of 1.5 MHz for a power density of about 30 mW/cm^2^. The appropriate location of that power density magnitude (10 to 100 mW/cm^2^) is shown below in Fig 7. The correlating diameter on the disk at which eg well tubes should be located in cell testing is 70.6 mm. Secondly, the ultrasound excitation on bacteria [26, 27] at a frequency of 28.5 kHz with power density of 500 mW/cm^2^ is shown also in Fig 7 for its magnitude (100 to 1,000 mW/cm^2^). The correlating diameter for well tubes for cell experiments is 103.1 mm.

**Fig 8:**
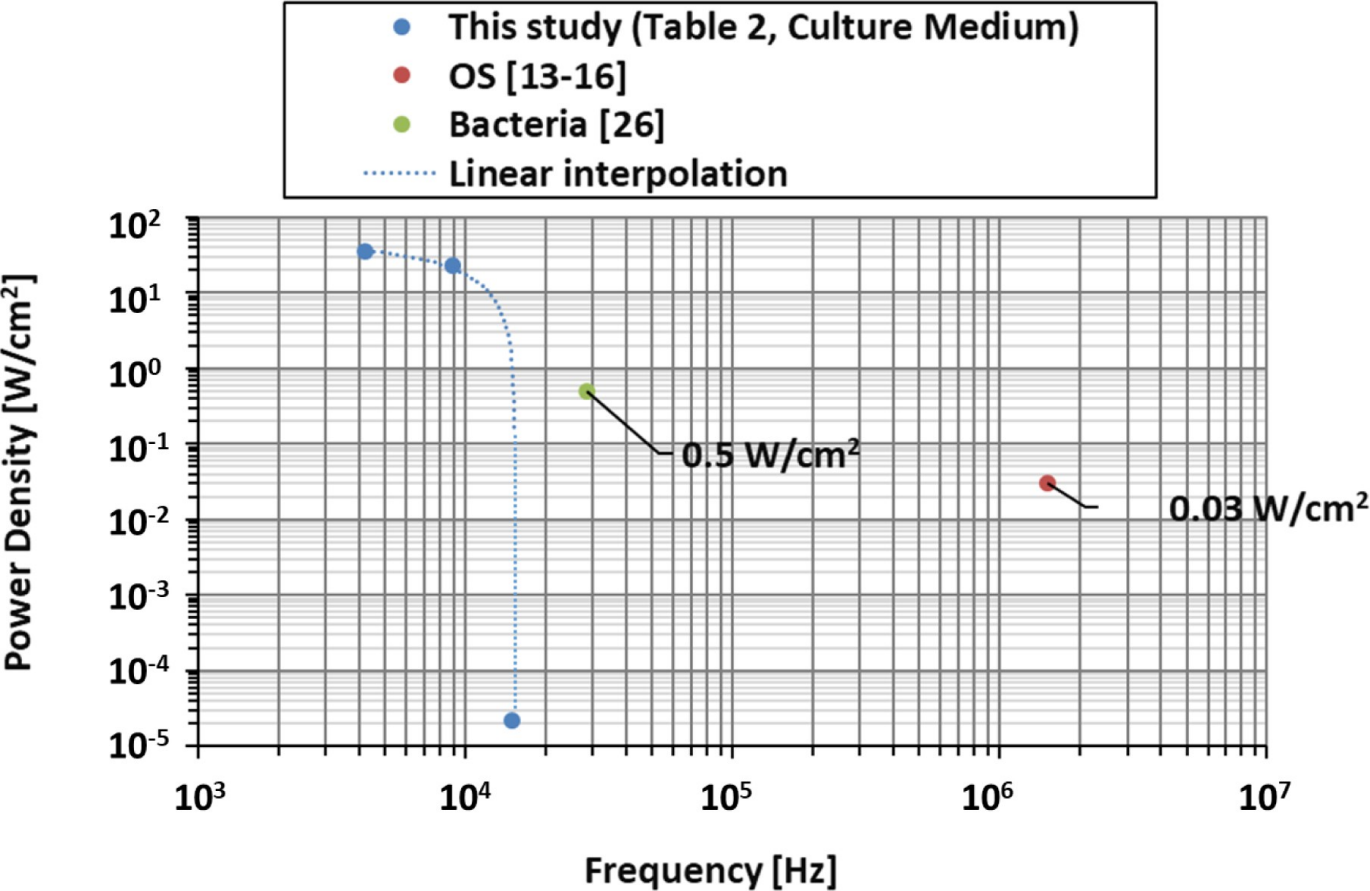
Comparison between power density of the vibration bioreactor in this study (4ND, 6ND, 8ND), OS studies, and bacterial studies.

The comparison of absolute values is shown in Fig 8. It can be seen that the power density in this study is greater for lower frequencies and that it can be as high as 70 times the ones tested on bacteria or 1,100 times the ones tested on OS. Bacteria and OS tests were performed with ultrasounds at higher frequencies that the ones applied in this study. The effect of the frequency on the cell behaviour under vibration has not been found in literature, but the vibration amplitude and power density presents a direct relationship with the cell behaviour as Deering et al. [34] showed recently in their study.

## 4. Conclusions and future work

After demonstrating the suitability of vibration mechanics for the design of vibration bioreactors [1], we investigate here further the power densities to which cell cultures are exposed. It is shown that our system is of greater suitability for a low frequency range below around 10,000 Hz where increasing power densities are obtainable. Our experiments were performed for water, sodium chloride 0.1N standard solution, and McCoy’s 5A w/L-Glutamine culture medium. The experimental results show a marginal decrease of natural frequencies and of resonance vibration amplitude once sodium is in the liquid, increasing liquid density. Numerical simulations based on the finite element method are performed and used to calculate local vibration power density. Experimental planning of cell testing in the future will have to consider that in resonance vibration the frequency is constant across the system while vibration magnitude varies. Suitable locations for well tubes are determined based on power density. A diameter of 70.6 mm for the magnitude of 10 to 100 mW/cm^2^ and a diameter of 103.1 mm for the magnitude of 100 to 1,000 mW/cm^2^ on the disk surface are identified. Useful next steps in future work [35] are the application of the bioreactors in actual cell experiments where cell cultures will be exposed to mechanical vibration. Possible effects which are achievable are cell destruction, enhanced cell proliferation, or antibacterial measures. Moreover, investigations on organ level are necessary. Those will serve to show how effects can be transferred from the petri dish onto eg bone geometries.
