# Correction: On the quantification of local power densities in a new vibration bioreactor

**DOI:** 10.1371/journal.pone.0248640

**Published:** 2021-03-10

**Authors:** David Valentin, Alexandre Presas, Charline Müntze-Röhr, Elisa Mele, Christoph Biehl, Christian Heiss, Wolfram A. Bosbach

The third author’s name is incorrect. The correct name is: Charline Müntze-Röhr. The correct citation is: Valentin D, Presas A, Müntze-Röhr C, Mele E, Biehl C, Heiss C, et al. (2021) On the quantification of local power densities in a new vibration bioreactor. PLoS ONE 16(1): e0245768. https://doi.org/10.1371/journal.pone.0245768

There are errors in the Author Contributions. The correct contributions are: Conceptualization: DV WAB. Data curation: DV. Formal analysis: AP DV. Funding acquisition: AP CH WAB. Investigation: DV AP. Methodology: AP. Project administration: WAB. Resources: CH. Software: DV, CM-R. Supervision: CB CH. Validation: EM CH WAB. Writing–original draft: DV WAB. Writing–review & editing: WAB.
